# Comparing opioid utilization and costs for surgical management of single-level spondylolisthesis: A national claims database analysis

**DOI:** 10.1016/j.jor.2024.06.012

**Published:** 2024-06-13

**Authors:** Hania Shahzad, Aziz Saade, Shannon Tse, Samuel K. Simister, Hamza Azhar, Hai Le, Safdar N. Khan

**Affiliations:** a Department of Orthopaedics, UC Davis Health, Sacramento, CA, USA; b The Ohio State University, Columbus, OH, USA

**Keywords:** Degenerative spondylolisthesis, Single-level, Lumbar, Decompression, Fusion, Instrumented fusion, Chronic opioid utilization, Healthcare costs

## Abstract

**Introduction:**

The rise in degenerative lumbar spondylolisthesis (DLS) cases has led to a significant increase in fusion surgeries, which incur substantial hospitalization costs and often necessitate chronic opioid use for pain management. Recent evidence suggests that single-level low-grade DLS outcomes are comparable whether a fusion procedure or decompression alone is performed, sparking debate over the cost-effectiveness of these procedures, particularly with the advent of minimally invasive techniques reducing the morbidity of fusion. This study aims to compare chronic opioid utilization and associated costs between decompression alone and decompression with instrumented fusion for single-level degenerative lumbar spondylolisthesis.

**Material and methods:**

Using data from the PearlDiver database, a retrospective database analysis was conducted. We analyzed records of Medicare and Medicaid patients undergoing lumbar fusion or decompression from 2010 to 2022. Patient cohorts were divided into decompression alone (DA) and decompression with instrumented fusion (DIF). Chronic opioid use, pain clinic visits, and total costs were compared between the two groups at 90 days, 1 year, and 2 years post-surgery.

**Theory:**

Does DIF offer a more cost-effective approach to managing DLS in terms of chronic opioid use in single-level DLS patients.

**Results:**

The study revealed comparable chronic opioid use and pain clinic visits between DA and DIF groups at 90 days and 1 year. However, total costs associated with opioid prescriptions as well as surgical aftercare were significantly higher in the DIF group at 90 days (p < 0.05), 1 year (p < 0.05), and 2 years (p < 0.05) post-surgery compared to the DA group.

**Conclusions:**

This study highlights the higher costs associated with DIF up to 2 years post-surgery despite comparable symptom improvement when compared to DA and DIF at the 1-year interval. DA emerges as a more financially favorable option, challenging the notion of fusion's cost-offsetting benefits. While further investigation is needed to understand underlying cost drivers and optimize outcomes, our findings emphasize the necessity of integrating clinical and economic factors in the management of single-level DLS.

## 1 Introduction

The prevalence of degenerative lumbar spondylolisthesis (DLS) has escalated over the past decade, primarily attributed to an aging population, necessitating surgical management when conservative treatments prove ineffective. In the United States, fusion rates for this condition have more than doubled from 2005 to 2014, with DLS accounting for a significant proportion of these procedures.^1^ The considerable hospitalization costs associated with lumbar internal fixation and fusion, estimated at $13 billion in 2011, surpass those of any other surgical procedure in the U.S^1^. The ongoing debate regarding the necessity of fusion surgery prompts a critical examination of its outcomes.^2^^,^^3^

Traditionally, while most surgeons have favored fusion surgery due to concerns about the long-term efficacy of decompression alone in addressing instability,^4^ they have failed to reach a consensus over a clear definition of instability.^5^ Recent evidence has emerged suggesting comparable outcomes between the two approaches in terms of symptom improvement, complications, and reoperations.^6^^,^^7^ In addition, the costs associated with implants and biologics,^8^ further contribute to the financial burden associated with fusion surgery.

Chronic opioid use is common among patients with chronic musculoskeletal pain,^9^ including those with DLS. Therefore, there is a growing interest in understanding the impact of surgical interventions on opioid utilization and associated costs in DLS patients. Due to its impact on quality of life, the financial implications of single-level DLS encompasses both direct costs associated with surgical and non-surgical treatments, as well as indirect costs related to lost productivity and ongoing patient care.^10^

The cost-effectiveness of adding fusion to decompression for the treatment of single-level degenerative lumbar spondylolisthesis remains uncertain. The emergence of less invasive, midline anatomy-sparing decompressive techniques has reignited interest in decompression alone for select patients.^11^ Conversely, the rising prevalence of lumbar fusions, accompanied by increased morbidity, mortality, and significant costs, has raised concerns regarding its clinical superiority.^12^

By investigating the cost-effectiveness of opioid utilization, pain management, and surgical aftercare following both approaches, we can determine whether the benefits of addition fusion to decompression outweigh its costs in the long term. This study aims to comprehensively explore and compare trends in chronic opioid utilization and associated costs of surgical aftercare between two surgical approaches for managing single-level degenerative spondylolisthesis, leveraging a large national claims database.

## 2 Methods

### 2.1 Study aims, setting and design

This study aimed to compare chronic opioid utilization and associated costs between surgical decompression alone and decompression with instrumented fusion for single-level degenerative lumbar spondylolisthesis. This study extracted patient records from the PearlDiver database, comprising over 92 million Medicare and Medicaid patients, with claims categorized using ICD-9 and ICD-10 codes. Procedures were classified based on Current Procedural Terminology (CPT) codes, while prescriptions and brand-name drugs were categorized using the Uniform System of Classification (USC) and the U.S. Food and Drug Administration National Drug Code Directory. This database was chosen due to its extensive patient population, minimizing the risk of Type-II errors. Ethical approval was not required as the database contains only deidentified patient records.

### 2.2 Eligibility criteria

Patient records were retrieved from the database based on a diagnosis of spondylolisthesis and lumbar fusion or decompression performed on the same day.^13^ Only records representing primary procedures were included, ensuring no prior fusion history. Multilevel fusion codes were excluded to focus solely on single-level procedures. Patient records who had any claims of opioid prescriptions within one month of the index surgery were also excluded to ensure that our population was opioid naïve. The patient cohort was then divided into two groups: those receiving decompression alone (DA) and those receiving decompression with instrumented fusion (DIF). Patients present in both cohorts were excluded from both groups. Data were reviewed for patient records coded between January 1, 2010, and October 31, 2022, identified through CPT codes.

### 2.3 Outcome measures

Our study focused on several key outcomes related to the surgical management of single-level spondylolisthesis, including chronic opioid use, visits to pain clinics at 90-day and 1-year intervals, and total costs at 90 days, 1 year, and 2 years. Chronic opioid use was defined as the presence of an interval of fewer than 30 days between two consecutive opioid prescriptions over 1 year. Pain clinic visits were identified using an in-built physician specialty filter for the pain department, and total costs were calculated using the database's built-in function for total costs at 90 days and 1 year, with results graphically presented. To assess the total cost, we first created a matched population of both surgical approaches based on age, gender, ECI score, insurance coverage plan, percentage of patients covered, and chronic opioid use. The total cost was determined using an inbuilt cost calculator within the database, intended to determine the cost of care for a patient associated with an existing event. This calculation included all costs within the defined period, and the resulting output provided the average reimbursement, as well as the distribution of reimbursements by percentile. The cost calculator begins a day after the surgery and therefore does not incorporate the costs of the surgery itself but rather only the post-operative management. Furthermore, we examined the trends in total costs associated with opioid prescriptions for the period 2010 to 2021 to provide additional context and insight into the financial implications of opioid utilization in the context of surgical management for single-level spondylolisthesis.

### 2.4 Statistical analysis

Independent student t-tests were conducted for continuous demographic variables, while chi-square analysis was employed for categorical variables. Additionally, multivariate regression analysis was performed to compare chronic opioid use and pain clinic visits after adjusting for age, gender, ECI, region, and insurance coverage. This statistical approach allowed us to control for potential confounding factors and assess the independent association of surgical management with these outcomes.

## 3 Results

### 3.1 Demographic characteristics

A total of 1649 patient records were retrieved, of which 255 (15.44 %) had undergone decompression alone (DA), while 1439 (87.22 %) had undergone decompression with instrumented fusion (DIF) (Table 1). The mean age of patients in the DA was 63.86 years, slightly higher than that of patients in the DIF group (61.59 years) with a statistically significant difference (p < 0.05). Gender distribution and ECI scores showed no significant difference between the two groups (p = 0.17, 0.16). A higher percentage of patients in the DIF were insured compared to the DA (90.11 % vs. 88.62 %, p < 0.05). Mean family income showed a nonsignificant trend toward higher income in the DIF compared to the DA (p = 0.11).

**Table 1:** Demographic characteristics of patients in Decompression Alone (DA) vs Decompression with Instrumented Fusion (DIF).

	Decompression Alone (n = 255)	Decompression & Instrumented Fusion (n = 1439)	P-value
Age (years)	63.86	61.59	<0.05
Gender			
*Males*	106	530	0.17
*Females*	149	909	
ECI^a^ score	5.35	5.00	0.16
Percentage of patients insured	88.62 %	90.12 %	<0.05
Mean family income^a^ ($)	81,000	83,000	0.11

a Elixhauser Comorbidity Index, Income rounded off to the nearest thousand.

### 3.2 Continuous opioid use

Multivariate regression analysis, adjusting for age, gender, ECI, plan, and region, using the DA group as the reference also showed no significant differences between both the groups in 90-day opioid use (aOR = 1.11, 95 % CI [0.66, 1.86], p = 0.70), 1-year opioid use (aOR = 0.74, 95 % CI [0.30, 1.81], p = 0.51), 90-day pain clinic visits (aOR = 1.31, 95 % CI [0.66, 2.59], p = 0.44), or 1-year pain clinic visits (aOR = 1.22, 95 % CI [0.77, 2.04], p = 0.42) (Table 2).

**Table 2:** Incidence of chronic opioid use and postoperative pain clinics in patients undergoing Decompression Alone (DA) vs Decompression with Instrumented Fusion (DIF) using multivariate regression analysis with DA as the reference group, adjusting for age, gender, ECI, insurance coverage, region.

	Decompression Alone (n = 255)	Decompression & Instrumented Fusion (n = 1439)	Odds Ratio [95 % CI]	P-value
*Chronic Opioid use*				

90-days	19	121	1.11 [0.66, 1.86]	0.70
1-year	6	28	0.74 [0.30, 1.81]	0.51

*Post-operative visits to pain clinics*				

90-days	11	79	1.31 [0.66, 2.59]	0.44
1-year	22	50	1.22 [0.77, 2.04]	0.42

*aOR-adjusted odds ratio, ^CI- confidence interval.

## 4 Cost burden

**Fig. 1:**
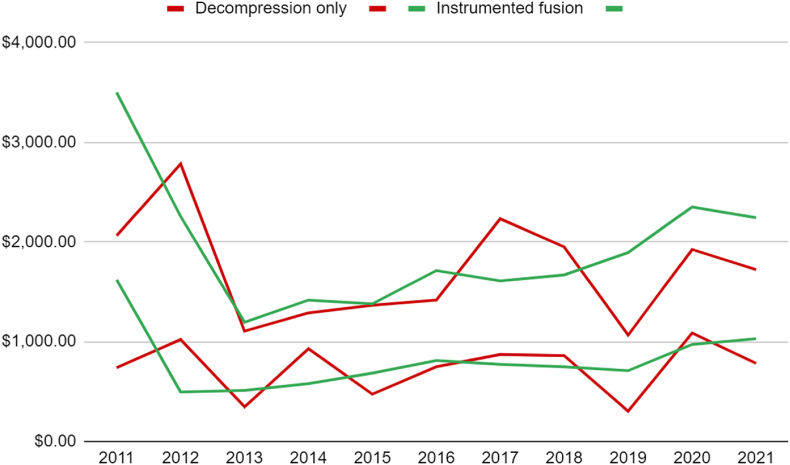
Total costs per patient attributed to opioid prescriptions at 90 days and 1 year between decompression only vs additional instrumented fusion.

For DA, the total costs per patient for opioid prescriptions at 90 days and 1 year fluctuated over the years, with values ranging from $300 to $1100 at 90 days and from $1100 to $1900 at 1 year (Fig. 1). In contrast, the costs associated with opioid prescriptions following instrumented fusion also exhibited fluctuations but generally trended higher compared to decompression alone. At 90 days post-surgery, the costs ranged from $500 to $1,600, while at 1 year, they varied from $1200 to $2400 (Table 3).

**Table 3:** 2-year total cost comparisons following surgery between both groups matched for age, gender, ECI, region, insurance coverage, and chronic opioid use.

Time	Decompression Alone ($)	Decompression & Instrumented Fusion ($)	P-Value
90-days	2000	11,000	<0.05
1-year	9000	17,000	<0.05
2-year	16,000	25,000	<0.05

All costs rounded off to the nearest thousand.

Total costs comparison between the two groups matched for age, gender, ECI score, insurance coverage plan, percentage of patients covered, and chronic opioid use, revealed significantly higher costs in the DIF group compared to the DA group at 90-days (p < 0.05), 1-year (p < 0.05), and 2-year (p < 0.05) post-surgery.

## 5 Discussion

This study aimed to address a pivotal question: in terms of opioid burden and its related costs, is adding fusion to the management of single-level degenerative spondylolisthesis beneficial? Our findings reveal that the improvement in symptoms, as reflected by chronic opioid use and visits to pain clinics, remains comparable between DA and DIF at the 1-year interval. Additionally, our investigation aimed to provide a two-year economic snapshot of the costs associated with post-surgical care following both procedures, demonstrating higher costs associated with instrumented fusion up until the 2-year mark.

Our results suggest that the symptom improvement, as indicated by chronic opioid use and post-operative specialty pain clinic visits, does not significantly differ regardless of the surgical approach. While the reliance on opioid prescriptions as a metric for symptom improvement is indirect, our findings align with previous studies that have compared these surgical approaches and found no significant difference in pain scores between decompression plus fusion and decompression alone.^2^^,^^3^^,^^14^, ^15^, ^16^, ^17^, ^18^ Although some studies have reported reduced leg pain scores in the fusion group, the heterogeneity of the baseline population has rendered this evidence controversial.^19^ Nevertheless, our study contributes to this body of evidence by demonstrating overall symptom improvement in terms of reduced opioid use, although the potential utilization of alternative pain management strategies, such as NSAIDs, warrants exploration.

The economic analysis, focusing solely on aftercare costs, suggests that decompression alone presents a more financially favorable option up to the two-year mark post-surgery. Contrary to previous notions suggesting that fusion's cost burden may be offset by lower rates of reoperation^12^^,^^20^ or enhanced symptom management compared to decompression alone, our findings challenge this assumption. It is important to note that the higher failure rate of decompression alone was noted in cases where a traditional laminectomy was performed, failing to preserve midline structures, and potentially leading to postoperative instability.^21^ In light of recent evidence, which suggests comparable outcomes and complication rates for both fusion and decompression for single-level degenerative spondylolisthesis, our study provides evidence for the economic benefits of decompression alone. However, further investigation is warranted to dissect the underlying factors contributing to the higher costs associated with fusion and to determine if these costs are preventable. Additionally, our study solely examined single-level degenerative spondylolisthesis cases, limiting the generalizability of our findings to cases involving higher levels or greater symptom severity, which may influence treatment decisions.

Comparing our results to previous studies, limited data are available regarding the economic benefits of either procedure in single-level degenerative lumbar spondylolisthesis. While some studies have assessed the cost-effectiveness of fusion versus decompression alone, caution is warranted due to the small sample sizes of DA cases in these analyses.^22^ For instance, Tosteson et al. reported varying cost per quality-adjusted life year (QALY) values, indicating that decompression alone had a lower cost per QALY compared to fusion surgery. Similarly, another cost-utility study concluded higher costs for instrumented fusion when comparing laminectomy alone to laminectomy with fusion.^23^^,^^24^ Thus, further research is needed to comprehensively evaluate the long-term cost-effectiveness and outcomes associated with these interventions.^4^

Despite the valuable insights provided by our study, it is not without limitations. We did not account for radiographic factors that may influence clinical decision-making, such as the specific level of spondylolisthesis (e.g., L4-L5) or the presence of stability or grade of spondylolisthesis. However, our study's strengths lie in being the first to investigate the cost implications and opioid use between these two surgical procedures while focusing exclusively on single-level procedures, where this clinical dilemma predominantly arises. Additionally, our study includes data from both neurosurgeons and orthopedic surgeons, utilizing a large multicenter dataset, thereby enhancing its generalizability.

## 6 Conclusion

In conclusion, our study delves into the debate surrounding the surgical management of single-level degenerative spondylolisthesis, specifically the benefit of adding instrumented fusion to decompression in terms of opioid burden and associated costs. Our findings indicate comparable symptom improvement between the two approaches at the 1-year mark, with chronic opioid use and pain clinic visits showing no significant differences. However, decompression alone emerges as a more financially favorable option, challenging the perceived cost-offsetting benefits of fusion surgery.

## Ethics approval and consent to participate

Not applicable. This study involved the analysis of deidentified patient records from the PearlDiver database, thus ethical approval and consent were not required.

## Consent for publication

Not applicable. This study did not contain any individual person's data in any form.

## Availability of data and materials

The datasets generated and/or analyzed during the current study are available from the corresponding author on reasonable request.

## Funding

This study did not receive any specific grant from funding agencies in the public, commercial, or not-for-profit sectors.

## Guardian/patient's consent

No patient consent was required as the data collected for this study were unidentified and did not involve any direct interaction with participants.

## CRediT authorship contribution statement

**Hania Shahzad:** Conceptualization, Methodology, Formal analysis, Writing – original draft. **Aziz Saade:** Writing – review & editing. **Shannon Tse:** Writing – review & editing. **Samuel K. Simister:** Writing – review & editing. **Hamza Azhar:** Data collection, Formal analysis, Data Interpretation, Writing – review & editing. **Hai Le:** Supervision, Writing – review & editing. **Safdar N. Khan:** Supervision, Project administration, Writing – review & editing.

## Declaration of competing interest

The authors declare that they have no competing interests.
